# Pathologic Characteristics of Patients in the Kamuzu Central Hospital Lymphoma Study (2013-2019)

**DOI:** 10.1200/GO.22.13000

**Published:** 2022-05-05

**Authors:** Morgan Dewey, Amy Lilly, Matthew Painschab, Tamiwe Tomoka, Yuri Fedoriw

**Affiliations:** ^1^ UNC Project Malawi, Lilongwe, Malawi; ^2^ The University of North Carolina at Chapel Hill, Chapel Hill, NC

## Abstract

We evaluated the concordance of lymphoproliferative disorder (LPD) diagnoses made initially at Kamuzu Central Hospital (KCH) with a limited immunohistochemistry panel and telepathology consultation with University of North Carolina (UNC) at Chapel Hill and second review at UNC with an extensive immunohistochemistry panel available.

**METHODS:**

The prospective KCH Lymphoma Study in Lilongwe has been enrolling patients with LPDs since 2013. Diagnoses are made by pathologists in Malawi and supported by US pathologists and Malawian clinicians at weekly telepathology conferences. Diagnostic tissue blocks are sent quarterly to UNC for secondary review and further confirmatory studies. Concordance of diagnosis from primary to secondary review was scored as: exact match (level 1), differences in granularity of the diagnosis (level 2), a change in classification but not in treatment (level 3), and a change to the diagnosis that would have altered the treatment course (Major Discordance). Cases with insufficient tissue for review at UNC were excluded from concordance evaluation.

**RESULTS:**

Three hundred eighty-eight adult patients were enrolled (June 2013-May 2019). Three hundred twenty-seven tissue blocks were sent to UNC for review and 292 LPDs diagnostic samples had sufficient quality for concordance review. The five most common LPDs were diffuse large B-cell lymphoma (n = 143, 49%), Burkitt lymphoma (9%, n = 25, 8%), classical Hodgkin lymphoma (8%, n = 23), Multicentric Castleman disease (8%, n = 23), and low grade non-Hodgkin lymphoma (6%, n = 17). Level 1, 2 and 3 concordance represented 175 (60%), 49 (17%) and 50 (17%) cases, respectively, and a Major Discordance was identified in 18 cases (6%).

**CONCLUSION:**

With a limited, selected immunohistochemistry panel and telepathology consultation, 94% of cases were accurately classified in real-time, leading to appropriate therapy. The evaluation of concordance provides a measure of quality assurance of the telepathology program, confidence in diagnoses for involvement in interventional trials, and useful data for understanding the epidemiology of LPDs in Malawi.

